# *In silico*-predicted B-cell epitopes for bovine brucellosis serodiagnosis: Preliminary analytical evaluation of synthetic peptide- and multi-epitope protein-based indirect ELISAs

**DOI:** 10.1371/journal.pone.0352788

**Published:** 2026-06-30

**Authors:** Lucas dos Reis de Souza, Monique Ferreira Silva Souza, Pâmela Aparecida Lima, Tatyane Martins Cirilo, Letícia Neves Ribeiro, Samuel Alexandre Pimenta Carvalho, Alessandro de Sá Guimarães, Patrícia Gomes de Souza, Paulo Martins Soares Filho, João Luís Reis Cunha, Lilian Lacerda Bueno, Tatiane Alves da Paixão, Ricardo Toshio Fujiwara, Renato Lima Santos

**Affiliations:** 1 Departamento de Clínica e Cirurgia Veterinárias, Escola de Veterinária, Universidade Federal de Minas Gerais, Belo Horizonte, Minas Gerais, Brazil; 2 Departamento de Patologia Geral, Instituto de Ciências Biológicas, Universidade Federal de Minas Gerais, Belo Horizonte, Minas Gerais, Brazil; 3 Departamento de Parasitologia, Instituto de Ciências Biológicas, Universidade Federal de Minas Gerais, Belo Horizonte, Minas Gerais, Brazil; 4 Empresa Brasileira de Pesquisa Agropecuária - Embrapa Gado de Leite, Juiz de Fora, Minas Gerais, Brazil; 5 Laboratório Federal de Defesa Agropecuária (LFDA-MG), Ministério da Agricultura e Pecuária, Pedro Leopoldo, Minas Gerais, Brasil; 6 University of York, York, United Kingdom; UFPL, BRAZIL

## Abstract

Serological methods are valuable diagnostic tools for bovine brucellosis, a zoonotic infectious disease with a worldwide distribution. Currently employed diagnostic methods utilize crude bacterial extract or regions of LPS, a molecule that can exhibit cross-reactions with other infectious agents. The goal of this study was to develop an indirect enzyme-linked immunosorbent assay (ELISAi) using synthetic peptides or a multi-epitope protein based on *in silico-*predicted B cell epitopes. Peptides were synthesized on a cellulose membrane using spot synthesis, and immunoblots were performed to evaluate reactivity by densitometry against bovine sera of interest. Peptides reacting with serum from positive cattle and non-reactive to the serum from negative controls were selected, synthesized in a soluble form, and used as antigens for the development of the ELISAi. Two peptides (P1 and P2) were selected and, after standardization of the ELISAi, positive (25) and negative (175) samples were tested, resulting in a sensitivity of 84% (21/25) and specificity of 83.43% (146/175). The sequence of the two peptides in replicates with spacers were inserted into a multi-epitope protein, which was also used as antigen in an ELISAi resulting in a sensitivity of 72% (18/25) and a specificity of 61.71% (108/175). This study provided a preliminary analytical assessment of *in silico*-predicted epitopes for developing novel serologic diagnostic tests.

## Introduction

Bovine brucellosis, historically known as contagious abortion or Bang’s disease, is a zoonotic and infectious disease with a worldwide distribution caused predominantly by *Brucella abortus*, and occasionally *Brucella melitensis* and *Brucella suis*. Infection by these last two species occurs mainly when there is shared management and rearing with small ruminants or pigs [1,2]. Bovine brucellosis is a disease of global concern due to significant economic losses and public health attention [3,4].

Serological methods have been widely used for the diagnosis of bovine brucellosis, commonly employing crude bacterial extracts or LPS as antigens [5]. The possibility of cross-reaction with other pathogens has been demonstrated and it is related to the immunogenic similarity of LPS from other gram-negative bacteria, such as *Yersinia enterocolitica* and *Escherichia coli* [6]. Endemic regions and those with herd health problems may present a high rate of false positives, thus impairing diagnostic and control measures [3]. In recent years, there has been a significant increase in research on new epitopes and methods for serological diagnosis of brucellosis. Many *Brucella* proteins have been recognized as having immunogenic and potential as antigens for diagnostic purposes, including the Bp26 [7], major outer membrane proteins (OMPs) [8], proteins of the VirB system [9]. The development of tests using protein components, such as immunogenic regions of OMPs [10], studies with synthetic peptides [11], and recombinant proteins [12], has demonstrated potential a diagnostic tools.

*In silico* prediction of B cell epitopes is based on the analysis of proteome sequences [13]. These epitopes can be predicted as linear or conformational forms and used for the development of vaccines, diagnostic methods, and immunotherapies [13]. Predicted epitopes can be synthesized as soluble peptides or on fixed platforms for *in vitro* reactivity assessment and subsequent use in diagnostic tests [14]. Therefore, the goal of this study was to develop an indirect enzyme immunoassay for the serological diagnosis of brucellosis in cattle using *in silico* predicted B cell epitopes with two distinct antigens: short synthetic peptides and a multi-epitope recombinant protein.

## Materials and methods

### Ethics statement

Serum samples employed in this study were from our archive and were obtained in previous studies [15] that had protocols approved by the Institutional Animal Care and Use Committee (Comissão de Ética no Uso de Animais/Universidade Federal de Minas Gerais – CEUA/UFMG – protocols # 204/2010; 197/2014; 56/2016; and 365/2018, and 157/2019). Bovine serum samples were also provided by the Empresa Brasileira de Pesquisa Agropecuaria (Embrapa Gado de Leite; CEUA protocol # 9804290621).

### Bovine serum samples

Twenty five bovine serum samples provided by the Federal Laboratory for Agricultural Defense (LFDA-MG) that were positive for brucellosis in the rose Bengal serological tests, slow tube agglutination (SAW), and 2-mercaptoethanol (2-ME) were used as positive controls (Table 1). Eighteen samples were used as negative controls, including 12 serum samples from cattle from a non-endemic region in the State of Santa Catarina, Brazil, certified as disease-free and unvaccinated, and six negative samples from the state of Minas Gerais, Brazil, an endemic region with vaccination. Samples from cattle seropositive for other pathogens were used to evaluate cross-reactivity. Twelve samples from cattle with concomitant infections by four pathogens other than *Brucella* sp., including *Neospora caninum* (seropositive by ELISA IDEXX kit 99–09566); *Leptospira* sp. (seropositive by Microscopic Agglutination Test – MAT); bovine herpesvirus type 1 (BHV-1) and bovine viral diarrhea virus – BVD (both seropositive by virus neutralization in MDBK cells, performed at the Instituto Biológico – São Paulo, Brazil). In addition, cattle with seroreactivity to individual pathogens were included as well: 19 samples positive for *Leptospira* sp. (12 samples from a brucellosis-free region without vaccination in the State of Goiás, Brazil, and seven from the State of Minas Gerais, all seropositive by MAT); 12 samples from calves positive for *Anaplasma* sp. (diagnosed by direct visualization of the pathogen in blood smears); nine samples positive for *Trypanosoma vivax* (diagnosed by direct visualization in smears of buffy coat); and two samples positive for *Neospora caninum* (seropositive by ELISA IDEXX kit 99–09566).

**Table 1:** Bovine serum samples used in the experiment.

Serum sample	Number of samples	Rose Bengal	SAW/2-ME	Vaccine	Origin	Age
Positive	25	Positive	Positive	Yes	MG-Brazil	>24 months
Negative controls	18	Negative	*	No (12)	SC-Brazil (12)	>24 months
			Negative	Yes (6)	MG-Brazil (6)	
Multiple infections	12	Negative	Negative	NI	MG-Brazil	>24 months
*Leptospira* sp.	19	Negative	*(12)	No (12)	GO-Brazil (12)	>24 months
			Negative (7)	Yes (7)	MG-Brazil (7)	
*Trypanosoma vivax*	9	Negative	*	NI	MG-Brazil	>24 months
*Neospora caninum*	2	Negative	*	NI	MG-Brazil	>24 months
*Anaplasma* sp.	12	Negative	Negative	No	MG-Brazil	<8 months
Vaccinated RB51	10	Negative	Negative	Yes (RB51)	MG-Brazil	<8 months
Vaccinated S19	22	Positive (6)	Positive (3)	Yes (B19)	MG-Brazil	6–18 months
		Negative (17)	Negative (20)			
Field samples	71	Negative	Negative	Yes (B19)	MG-Brazil	>24 months
*Total*	200					

*Test not performed due to insufficient sample volume.

Sera from cattle vaccinated with the RB51 vaccine strain (n = 10), obtained at 45 days and 3 months post-vaccination, were used to evaluate cross-reactivity with RB51-elicited antibodies. Sera from cattle vaccinated with the S19 vaccine strain, obtained at 3 months post-vaccination (n = 12) and 12 months post-vaccination (n = 10), were provided by the Empresa Brasileira de Pesquisa Agropecuária (EMBRAPA Gado de Leite – MG) and used to evaluate cross-reactivity with S19-elicited antibodies. Samples collected at 3 and 12 months were not from the same animals.

Samples were tested using the rose Bengal test, followed by SAW and 2-ME when sufficient volume was available (Table 1). The number of samples varied in individual experiments since some samples became unavailable over the course of this study.

### Selection of immunogenic epitopes of *Brucella* spp

Selection of immunogenic epitopes was based on the analysis of predicted proteomes of *B. abortus* (strain 2308 and bj1), *B. suis* (strain QH05 and 1330), *B. canis* (ATCC 23365 and GB1), *B. ovis* (ATCC 25840) and *B. melitensis* (CIT21 and bv 1 str 16M) as previously described [15].

### Spot synthesis

In order to assess reactivity of predicted epitopes (synthetic peptides), they were synthesized and spotted on cellulose membranes using an automated synthesizer (ResPepSL/Automatic Spot Synthesizer) [16]. FMOC amino acids (Novabiochem) were used, and couplings were performed through cycles by adding 25% 4-methylpiperidine, which removes the FMOC fragment, allowing the addition of the next amino acid [17].

#### Immunoblotting.

The membrane containing the synthetic peptides was subjected to immunoblotting for screening reactive epitopes. Initially, the membrane containing all predicted peptides was exposed to pooled serum from positive (n = 8) or negative (n = 8) cattle, and the peptides reactive to bovine serum were selected and synthesized on a new membrane. After test optimization, the peptides on the membrane were exposed to pooled serum from positive (n = 8), negative (n = 8), vaccinated with RB51 at 45 days after vaccination (n = 8) or at three months after vaccination (n = 8); vaccinated with S19 at three months after vaccination (n = 8) or at 12 months after vaccination (n = 8), *Leptospira* sp. positive samples (n = 8) and serum samples from cattle infected by pathogens other than *Brucella* sp. (*Neospora caninum* – n = 2; *Trypanosoma vivax* – n = 2; *Anaplasma* sp. - n = 2; and with multiple infections – n = 4). The membrane was blocked with a solution containing 5% bovine serum albumin (BSA) and 4% sucrose in 1x PBS for 12–16 hours (overnight) at room temperature and under agitation at 60 RPM. After the blocking period, the membrane was washed three times with a washing solution (1x PBS + 0.1% Tween 20) under agitation at 60 RPM for 10 minutes each wash, and then incubated for two hours with serum samples at dilutions of (1:1000). After incubation, the membrane was washed three times as described above, and incubated for one hour with an anti-bovine IgG secondary antibody (Sigma-Aldrich, St. Louis, USA) diluted in the washing solution at a ratio of (1:100,000) at room temperature at 60 RPM. After incubation with the secondary antibody, the membrane was washed as described above. For reading the results, a dark tray for luminescence reading was used, adding approximately 3 mL of luminol (Luminata Forte Western HRP substrate – Merck, USA) across the entire membrane. The spots were detected using an ImageQuant LAS 4000 reading device (GE Healthcare Life Sciences), with an exposure time of one minute for image capture [18,19]. After analysis, the membrane was regenerated and reused in subsequent analyses.

### Selection of epitopes for the synthesis of soluble synthetic peptides

After immunoblotting with the sera of interest, reactive peptides were analyzed by densitometry using the ImageJ software (Protein Array Analyzer). The average background signal on the membrane was subtracted from the intensity of each spot. The cut-off was calculated using the average of the four least reactive spots on the membrane exposed to the negative control serum pool plus twice the standard deviation of this average. Target peptide selection was based on the specificity and reactivity of the spots, excluding reactive peptides in the negative control serum, those that reacted with serum from animals infected by other agents, vaccinated animals, and non-reactive or weakly reactive spots in the positive serum. The selected synthetic peptides were then synthesized as soluble peptides using an automated synthesizer (ResPepSL/Automatic Spot Synthesizer, Intavis GmbH, Cologne, Germany), with FMOC amino acids (Novachem) and Rink Amide resin (Sigma) [20]. The test tubes containing the synthetic peptides were weighed and hydrated in Milli-Q water in sufficient quantity to achieve a concentration of 5 μg/μL.

### ELISAi using soluble synthetic peptides as antigen

Synthetic peptides were used as antigens in the Indirect Enzyme-Linked Immunosorbent Assay (ELISAi) technique. The assays were performed in 96-well polystyrene plates (COSTAR 3590 EIA/RIA PLATE). To standardize the technique, tests were conducted evaluating several variables, such as antigen concentration on the plate at concentrations of 0.5, 1.0, and 2.0 μg/well, bovine serum dilution (1:25, 1:50, 1:100, 1:200, 1:400, 1:600, and 1:800), bovine anti-IgG secondary antibody dilution (1:2500, 1:5000, 1:10000, 1:20000, and 1:40000), and development time (10 min and 25 min). The tests were performed with a minimum of 10 positive and negative control samples. The parameters that showed the best ratio between the positive and negative control samples were considered ideal.

After standardization of the assay, the peptides were individually tested against positive and negative control sera. The selection criteria were based on the ROC curve analysis, considering sensitivity, specificity, and area under the curve (AUC). The ratio between the mean optical densities (OD) of the positive and negative control groups was also considered. Peptides with the best results were selected to be used in pooling for further tests, and various peptide pool combinations were evaluated. The peptide combination that showed the best results based on the selection criteria was used in the development of the final ELISAi test. Plates were sensitized with the peptides at a concentration of 1.0 μg/well and incubated in an oven at 37°C for 12 hours. After sensitization, three washes were performed with 1x PBS + 0.05% Tween 20 (200 μL per well), followed by blocking of nonspecific reactions with 5% BSA solution in 1x PBS (200 μL per well) for 2 hours at 37°C. The sera of interest were diluted (1:50) in 2.5% BSA solution in 1x PBS and incubated at 37°C for 1 hour (100 μL per well). Plates were washed as described above, and the secondary antibody (anti-bovine IgG conjugate) diluted (1:10,000) in 2.5% BSA solution was added and incubated for 1 hour at 37°C (100 μL per well). Plates were washed and a developing solution containing citrate/phosphate buffer (pH 5.0), 0.05% o-phenylenediamine dihydrochloride (OPD), and 0.1% hydrogen peroxide (100 μL of developing solution per well) was added. The plate was incubated in the dark for 10 minutes, and the reaction was stopped by adding 2N sulfuric acid (50 μL per well). The resulting absorbance was measured using an ELISA reader (MR-96a Microplate reader at 492 nm) [21]. All reactions were evaluated in duplicate, with negative (pool of ten negative serum samples), positive (pool of ten positive serum samples), and blank controls (wells without test serum sample).

### Production of a multi-epitope protein

The nucleotide sequences of the selected peptide 1 (PAPKAKPKPPQAQTA) and peptide 2 (PRRTAAKDSGDDTPV) were used to generate a recombinant multi-epitope protein. The sequences were arranged in 10 replicates interspersed with GKGK (Glycine-Lysine-Glycine-Lysine) linkers. Methionine from the vectors themselves was used to initiate protein synthesis, and in the case of the expression vector, and a histidine tag was inserted at the N terminal portion of the protein for subsequent purification by nickel columns and identification by Western blot (S1 Fig).

### Bacterial Transformation and expression of the multi-epitope protein

The pUC57 plasmid (GenScript, Nanjing, China) containing the multi-epitope protein synthetic gene was transformed into *E. coli* (XL1_Blue) by electroporation (Bio-Rad, USA). Transformation was confirmed by selective growth on 2xYT Agar medium with ampicillin (100 μg/mL) incubated at 37°C for 12–16 hours. Colonies growing in the antibiotic medium were individually selected and cultured in liquid 2xYT medium with ampicillin (100 μg/mL) at 37°C under agitation at 180 rpm for 12–16 hours, and then processed for plasmid DNA extraction (PureLink QuickPlasmid Miniprep Kit; Invitrogen, Carlsbad, USA). The synthetic gene was then subcloned into the expression vector pET-28a(+)-TEV, using the restriction enzymes *Nhe*I and *Xho*I and standard cloning techniques. The pET-28a(+)-TEV/gene construct was used for electrotransformation of expression bacteria in the Shuffle T7 strain.

For protein expression bacteria were cultured in 50 mL of 2xYT liquid medium with kanamycin (50 μg/mL) at 30°C with agitation at 180 rpm for 12−16 hours, then 50 mL of bacterial suspension was subcultured in 1 L of 2xYT liquid medium with kanamycin (50 μg/mL) under the same conditions until an OD between 0.6 and 0.8 was reached. Subsequently, 1 mM Isopropyl B-D-1-Thiogalactopyranoside (IPTG) was added, and the culture was maintained at 30°C under agitation (180 rpm) for 4 hours, and then centrifuged for 30 minutes at 2,000 x *g* at 4°C. The bacterial pellet was resuspended 30 mM imidazole in 1x PBS solution, and lysed with lysozyme (100 μg/mL; Sigma-Aldrich, USA) on ice for 1 hour using a high-pressure EmulsiFlex-C3 homogenizer (AVESTIN, Canada) and subsequently centrifuged at 10,000 x *g* at 4°C for 1 hour, and the supernatant was filtered with 0.45 μm filters. The filtered supernatant was purified by ion affinity chromatography using an ÄKTAprime plus (GE Healthcare Life Sciences, USA) purifier and nickel columns (HisTrap FF 5 mL - Sigma-Aldrich). Protein fractions were identified and the protein concentration estimated by Kit Pierce BCA Protein Assay (Thermo Fisher Scientific, USA), according to the manufacturer’s instructions. Protein expression was confirmed by evaluation using polyacrylamide gel electrophoresis (SDS-PAGE) with assessment of intensity and reference to a molecular standard to estimate the molecular mass of the fractions and by anti-6xhis monoclonal antibody (GE Healthcare Life Sciences, United Kingdom) Western blot (S1 Fig), as previously described by Souza and colleagues [15].

### ELISAi with recombinant multi-epitope chimeric protein

ELISAi with multi-epitope protein was performed using 96-well flat polystyrene plates. Standardization was performed following criteria similar to the ELISAi with synthetic peptides. After standardization, the ELISAi was performed with the serum sample group using the protein at a concentration of 100 ng/well to sensitize the plates with dilution in carbonate/phosphate buffer (pH 9.5) in a refrigerator for 12–16 hours. Blocking was performed with a 5% BSA solution for one hour in an incubator at 37°C, followed by the addition of sera diluted 1:100 in 2.5% BSA for one hour in an incubator at 37°C. The secondary anti-bovine IgG antibody was diluted 1:10,000 in 2.5% BSA for one hour at 37°C, and development was performed with a developing solution containing citrate/phosphate buffer (pH 5.0), 0.05% o-phenylenediamine dihydrochloride (OPD), and 0.1% hydrogen peroxide (100 μL of developing solution per well). The plate was incubated in the dark for 10 minutes, and the reaction was stopped by adding 2 N sulfuric acid (50 μL per well). The resulting absorbance reading was performed using an ELISA reader (MR-96a Microplate reader at 492 nm) [21]. All reactions were evaluated in duplicate, with negative, positive, and blank controls.

### Statistical analysis

Data were organized and analyzed using Microsoft Excel, and graphs were generated using GraphPad Prism 8.01 software (GraphPad, USA). ELISAi cutoff points were determined based on the best sensitivity-specificity ratio using the ROC (Receiver Operating Characteristic) curve. Kappa analysis was used to assess the agreement between the results of the developed ELISAi and rose Bengal using MedCalc Statistical Software version 20.2.

## Results

### *In silico* prediction and selection of epitopes

**Fig 1:**
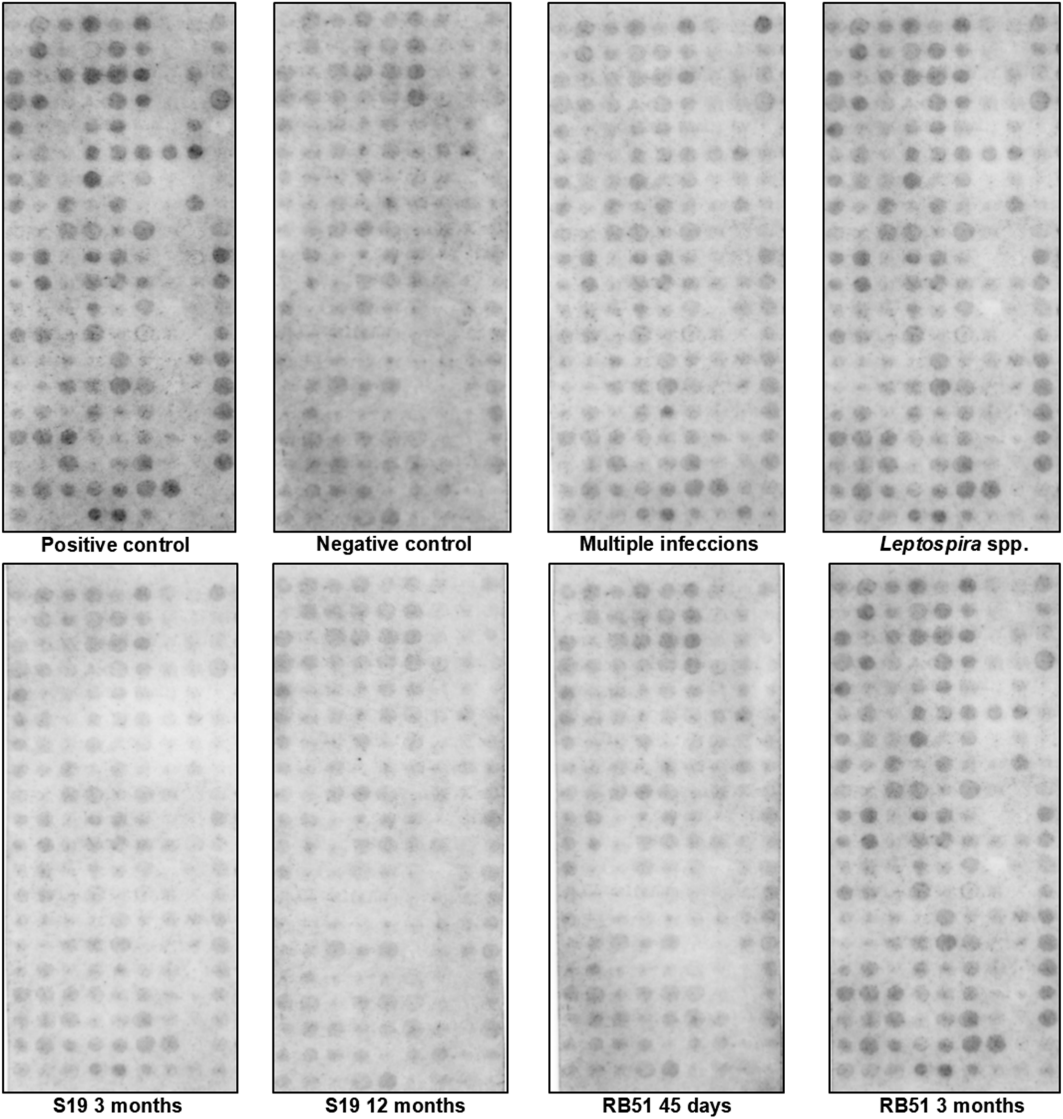
Immunoblotting in a spot membrane. Each image represents an individual analysis of the membrane exposed to bovine serum pools: positive control (n = 8), negative control (n = 8), with multiple infections (*Neospora caninum* – n = 2; *Trypanosoma vivax* – n = 2; *Anaplasma* sp. - n = 2; and with multiple infections – n = 4)*, Leptospira* sp. infection (n = 8), vaccinated with S19 collected 3 and 12 months post-vaccination and vaccinated with RB51 with samples collected 45 days (n = 8) or at 3 months post-vaccination (n = 8). Darker spots are more reactive to the bovine serum used. Images obtained using ImageQuant LAS 4000, luminescence technique.

**Fig 2:**
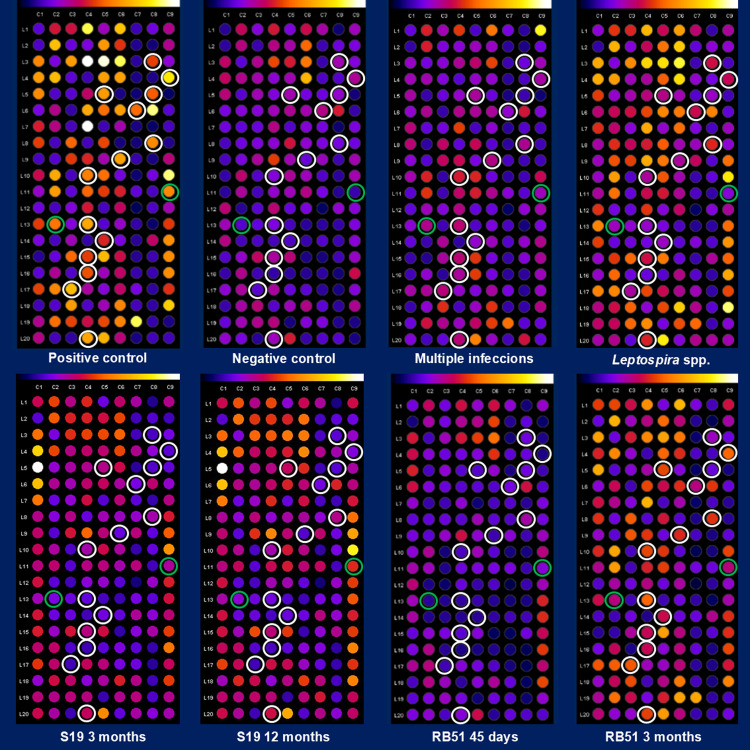
Densitometry of the cellulose membrane with the 16 pre-selected peptides in the immunoblotting technique. Each membrane represents an individual analysis with a pool of serum samples of interest. Spots with higher densitometry values are white, and those with lower values are dark blue (representing the intensity indicated by the colored bar above the spots). The 16 pre-selected peptides in the analysis are circled in white, and the two peptides selected for the development of the final ELISAi and multi-epitope recombinant chimeric protein are in green. Cut-off of 14640.81, density values obtained for the selected peptides are available in S1 Table.

A total of 571 peptides were predicted *in silico* as B cells epitopes, including species-specific or genus-specific peptides (Table 2). Peptides were synthesized on a cellulose membrane to evaluate their reactivity against bovine serum positive and negative for brucellosis. At this initial stage, 180 (out of the original 571) peptides that were reactive to bovine serum were selected; the remaining peptides (391/571) were discarded from the analysis, and a new membrane with these 180 reactive peptides was synthesized. The membrane containing 180 peptides was exposed to pools of various bovine serum groups (Fig 1). Using densitometry analysis with ImageJ (Protein Array Analyzer) software, a cutoff of 14640.81 was obtained, and sixteen peptides were selected for synthesis in their soluble form (Fig 2). Densitometry values and cutoff points of the selected peptides are shown in S1 Table. Peptides exposed to the positive serum pool with a densitometry value below 14640.81 were excluded due to low reactivity, as were peptides with reactivity values higher than 14640.81 when exposed to the negative pool, sera from vaccinated animals, or sera from animals infected with other agents.

**Table 2:** Number of genus-specific or species-specific peptides (*in silico*-predicted B cell epitopes).

Species	Number of peptides
*Brucella* spp. (conserved in all species)	421
*B. abortus*	38
*B. melitensis*	51
*B. suis*	7
*B. ovis*	44
*B. canis*	9
Smooth species (*B. abortus* e *B. suis*)	1
Total	571

### ELISAi with synthetic peptides

The 16 soluble peptides were subjected to ELISA tests, evaluating their individual diagnostic capacity against positive (n = 19) and negative (n = 21) sera (S2 Fig). The selection of the best peptides considered the results of the ROC curve analysis, such as sensitivity, specificity, and area under the curve (S2 Table). Peptides P1, P2, and P7 were selected after analysis of the ELISA results and used in further tests as pools. Assays were performed using the same serum samples previously tested with pools of P1 + P2, P1 + P7, P2 + P7, and a pool of P1, P2, and P7 (S3 Fig). The selection of the best peptide combination considered the results of the ROC curve analysis (S2 Table). The pool of P1 and P2 was considered the best based on the results of the ROC curve analysis and the OD ratio.

**Fig 3:**
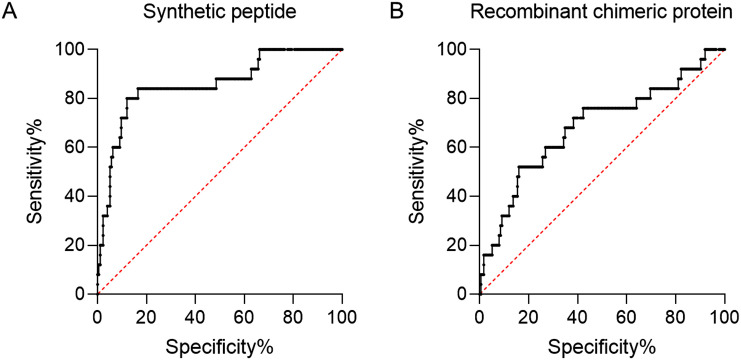
Receiver Operating Characteristic (ROC Curve) analysis of the ELISAi of synthetic peptides and multi-epitope recombinant chimeric protein. **(A)** ROC curve of the ELISAi of synthetic peptides with an area under the curve of 0.8565 (95% CI = 0.7703–0.9426) and p < 0.0001. **(B)** ROC curve of the ELISAi of recombinant protein with an area under the curve of 0.6841 (95% CI = 0.5618 - 0.8065) and p < 0.01.

**Fig 4:**
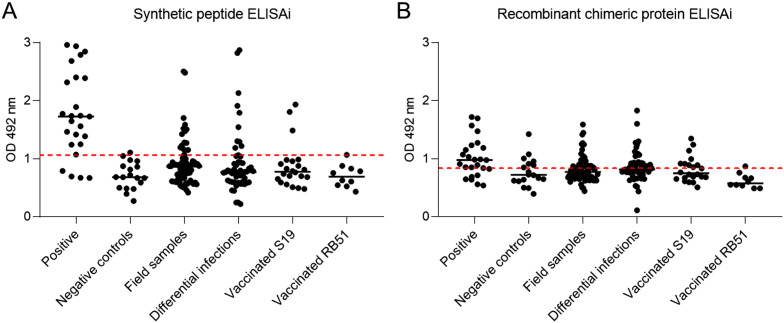
Representation of the results in the ELISAi of synthetic peptides and multi-epitope recombinant chimeric protein with individual bovine serum samples. (A) Synthetic peptide ELISAi, the cut-off calculated based on ROC curve analysis was 1.070 (red line), sensitivity of 84% (95% CI = 65.35 - 93.60%) identifying 21/25 positive samples and specificity of 83.43% (95% CI = 77.21 - 88.21%) identifying 146/175 negative samples. The false positive samples were respectively 1/18 negative control sample, 15/71 field samples, 10/54 samples from infections by other agents and 3/22 recently vaccinated with S19. **(B)** For recombinant protein ELISA, the cut-off calculated based on the ROC curve was 0.8372 (red line) with a sensitivity of 72% (95% CI = 52.42–85.72), identifying 18/25 positive samples, and a specificity of 61.71% (95% CI = 54.33–68.59%), correctly identifying 108/175 negative samples. False positive samples were 7/18 negative controls, 27/71 field samples, 23/54 differential infections, 9/22 recently vaccinated with S19, and 1/10 vaccinated with RB51. Each point represents an individual sample.

**Fig 5:**
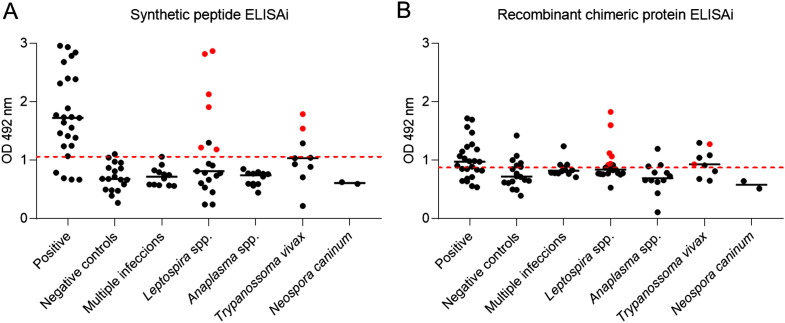
Representation of the ELISAi of synthetic peptides and multi-epitope recombinant chimeric protein using serum samples with differential *Brucella* infections. **(A)** Synthetic peptide ELISAi: serum samples with infections by other agents that reacted falsely were 7/19 *Leptospira* sp. samples and 3/9 *Trypanosoma viv*ax samples; the red dots represent samples with false positive results in both tests. **(B)** Recombinant protein ELISAi: serum samples with infections by other agents that reacted falsely were 4/12 infections by multiple agents, 10/19 *Leptospira* sp., 3/12 *Anaplasma* sp., and 6/9 *Trypanosoma vivax*. The dots represent individual samples.

The P1 and P2 pool was employed as antigen for developing the ELISAi under the best analytical conditions, and 200 bovine serum samples were then evaluated. The ROC curve had an area under the curve of 0.8565 (95% CI = 0.7703–0.9426) and p < 0.0001 (Fig 3A). The cut-off calculated based on the ROC curve analysis was 1.070, with a sensitivity of 84% (95% CI = 65.35–93.60%) identifying 21/25 positive samples and a specificity of 83.43% (95% CI = 77.21–88.21%) identifying 146/175 of the negative samples (Fig 4A). Twenty-nine samples were false positives, of which these one was a positive control from a disease-free region without vaccination, seven samples with infection by *Leptospira* sp., three samples from cattle infected with *Trypanosoma vivax* (Fig 5A), three from cattle recently vaccinated with S19, and 15 negative samples. Kappa analysis showed moderate agreement (0.4153 CI95% = 0.2658–0.5668) between the peptide ELISAi and rose Bengal.

### ELISAi with multi-epitope protein

The ELISAi with the multi-epitope protein resulted in an area under the ROC curve of 0.6841 (95% CI = 0.5618–0.8065; p < 0.01) (Fig 3B). The cut-off calculated based on the ROC curve was 0.8372 with a sensitivity of 72% (95% CI = 52.42–85.72), identifying 18/25 positive samples, and a specificity of 61.71% (95% CI = 54.33–68.59%), correctly identifying 108/175 negative samples (Fig 4B). Four out of 29 of the samples that were false positive in the ELISAi with peptides had a negative result in this test; whereas 42 new samples were false positive, including mostly field samples and those with infections by other agents (Fig 5B). Kappa analysis showed no agreement between the protein ELISAi and rose Bengal (Kappa = 0.1620, 95% CI = 0.049–0.275).

## Discussion

*In silico* epitope prediction for the serological diagnosis of *Brucella* spp. infection in cattle, followed by the production of soluble antigens, yielded an analytical sensitivity of 84% and specificity of 83% in an ELISAi, which had moderate agreement with rose Bengal. Conversely, the multi-epitope protein had reduced sensitivity and specificity when compared to synthetic peptides. Previous studies demonstrate that the production of a multi-epitope protein can increase sensitivity and specificity when compared to the same short linear epitopes [22,23] or provide similar results [15], which contrasts with this study. Despite the evaluation of solubility status and conformational stability by bioinformatics and confirmation of protein characteristics by PCR and western blot, protein production in vectors and prokaryotic cells may result in unpredictable conformation that may impair antibody recognition, including truncated or misfolded proteins, which may escape prediction by the bioinformatics analysis [24]. Furthermore, another hypothesis is that the strategy of using a repetitive design including 10 replicates with GKGK linkers may have promoted steric masking, altering epitope accessibility or producing a non-native antigenic presentation.

The use of synthetic peptides selected by *in silico* prediction can generate greater specificity due to the selection of target antigens [15,14]. However, in this study, samples from animals infected with *Leptospira* sp. and *Trypanosoma vivax* resulted in false positive results. Cross-reactivity with leptospirosis is a known condition that can occur in animals with natural infections or vaccinated cattle, leading to false-positive results as previously reported [25]. Cross-reactivity of *Brucella* antigens with *Leptospira* antibodies have also been documented in *B. canis*-infected dogs [26]. In contrast, cross-reactivity with sera from *Trypanosoma*-infected animals is not commonly reported. Despite an stringent screening aimed to minimize cross-reacting antigens, the peptides selected in this study showed a non-specific reaction with sera from *T. vivax*-infected cattle. A plausible hypothesis is that cross-reactivity in this case may be due to conformational (nonlinear) epitopes, which were not detected by genomic or proteomic analyses. Therefore, although our initial assessment of linear amino acid sequence similarity suggested that they are not conserved in *Leptospira* sp. and *T. vivax*, we observed some level of cross reactions with those pathogens so in areas where these diseases overlap, there might be a need for secondary tests to discriminate between these infections.

Positive results in three animals vaccinated with S19 in the ELISAi with synthetic peptides were expected, given that vaccine reactivity has already been detected in other diagnostic tests [6]. The field cattle with false-positive results in the ELISAi were mostly vaccinated with S19, and despite being older than 24 months, it was not possible to determine whether vaccine antibodies, even in small quantities, could induce a positive result in the tests developed in this study. Application to samples with distinct epidemiological contexts, such as a vaccination-free area, is necessary for a better understanding of this variable.

The use of recombinant chimeric proteins and synthetic peptides has demonstrated good diagnostic capability in brucellosis, with various methods employed, ELISA being one of the main ones, both in the diagnosis of the disease in humans and animals [27,28,29,9,23], but recombinant proteins or synthetic peptides are also used in immunochromatographic tests [11], dot blots [30] and other serological diagnostic techniques, and the antigens developed here can be used in other diagnostic methodologies.

The use of a synthetic antigen allows for greater batch uniformity, since crude antigen used in rose Bengal, 2-ME and SAW tests must always be standardized and undergo conformity tests whenever a new batch needs to be produced, and studies have shown that there may be differences in the diagnostic capacity of rose Bengal antigens between batches and regions of the world [31].

Several limitations should be considered when interpreting our findings. First, the performance estimates were derived from only 25 positive samples, resulting in substantial statistical uncertainty and consequently wide confidence intervals. Although these intervals were reported, their implications for the robustness and generalizability of the results were not adequately addressed. Second, the discovery and evaluation phases were conducted using overlapping sample sets, raising the possibility of overfitting and leading to potentially optimistic estimates of diagnostic performance. Finally, independent validation in a larger, external cohort is essential before any conclusions regarding the clinical or diagnostic utility of the proposed approach can be considered reliable. Yet, our study reaffirms that *in silico* prediction for selecting new epitopes is useful for developing diagnostic tests. This study provided a preliminary analytical assessment of methods so our results should be considered a proof-of-concept of the feasibility of *in silico* epitope predictions for developing novel serologic diagnostic tests.

## Supporting information

S1 Fig Western blot anti-His tag of purified fractions with protein concentration obtained from *E. coli* Shuffle. The fractions show a strongly labeled band at a height between 50 and 75 kD.
(TIF)

S2 Fig Results of the ELISAi of individual synthetic peptides. Data are presented as individual values for each positive control (n = 19) and negative control (n = 21) sample. The dashed line represents the cut-off point of each analysis, and the bar represents the mean OD of each group (Sensitivity, specificity, and OD ratio results are available in S2 Table). Peptides PEP1 (A), PEP2 (B), and PEP7 (G) were selected for pool based on the ROC curve results. All analyses were performed in duplicate, and the values represent the mean OD of each sample.
(TIF)

S3 Fig Results of the ELISAi with pool peptides PEP1, PEP2, and PEP7. Data are presented as individual values for each positive (n = 19) and negative (n = 21) control sample. The dashed line represents the cutoff point for each analysis, and the bar represents the median OD of each group. A) PEP1 + PEP2 peptide pool showed superior results to the others based on ROC curve analysis, with sensitivity (84.21% − 95% CI = 62.43–94.48), specificity (95.24% − 95% CI = 77.33–99.76), and AUC (0.9373–95% CI = 0.8644–1.0) p < 0.0001. Sensitivity, specificity, and OD ratio results are available in S2 Table. All analyses were performed in duplicate, and the values represent the mean OD of each sample.
(TIF)

S1 Table Densitometry values obtained for each selected peptide in the spot membrane immunoblotting technique. The columns represent the selected peptides P1-P16 and the densitometry values obtained in the sample pools of positive control, negative control, *Leptospira* spp., multiple infections, vaccinated with the RB51 vaccine at 45 days (45d) and 3 months post-vaccination, and vaccinated with S19 at three months (3m) and twelve months (12m) post-vaccination. Cut-off of 14640.81.
(PDF)

S2 Table Results of ROC curve analysis and OD ratio (positive control/negative control) of ELISAi of individual and pool peptides. p-value <0.0001 for all analyses. (PDF)
