# Supplementary figures and images for: In silico-predicted B-cell epitopes for bovine brucellosis serodiagnosis: Preliminary analytical evaluation of synthetic peptide- and multi-epitope protein-based indirect ELISAs
Source: PLoS One. 2026 Jun 30;21(6):e0352788. doi: 10.1371/journal.pone.0352788 (PMC13318027; doi:10.1371/journal.pone.0352788)

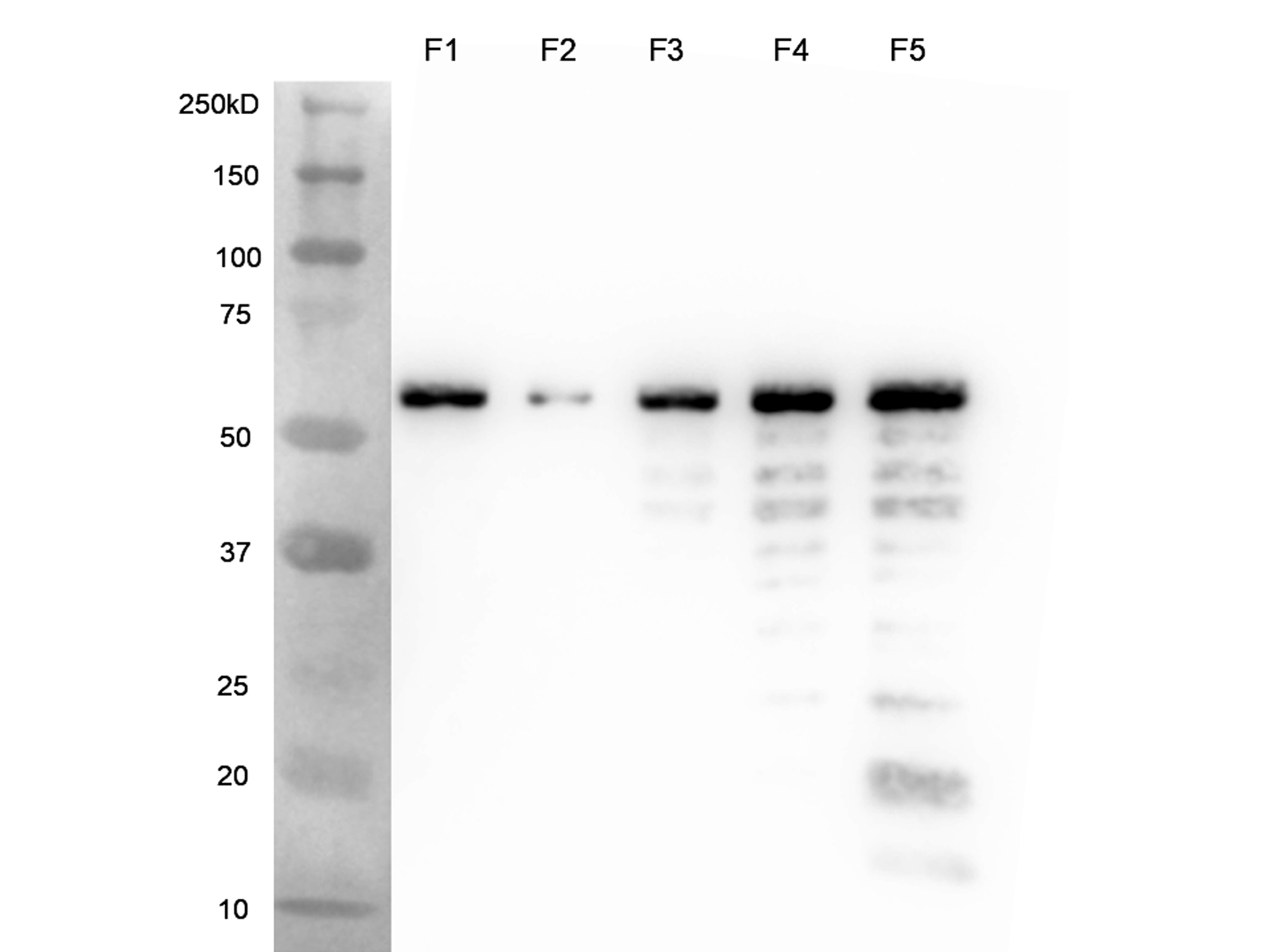

Supplement: S1 Fig — The fractions show a strongly labeled band at a height between 50 and 75 kD. (TIF) [file pone.0352788.s001.tif]

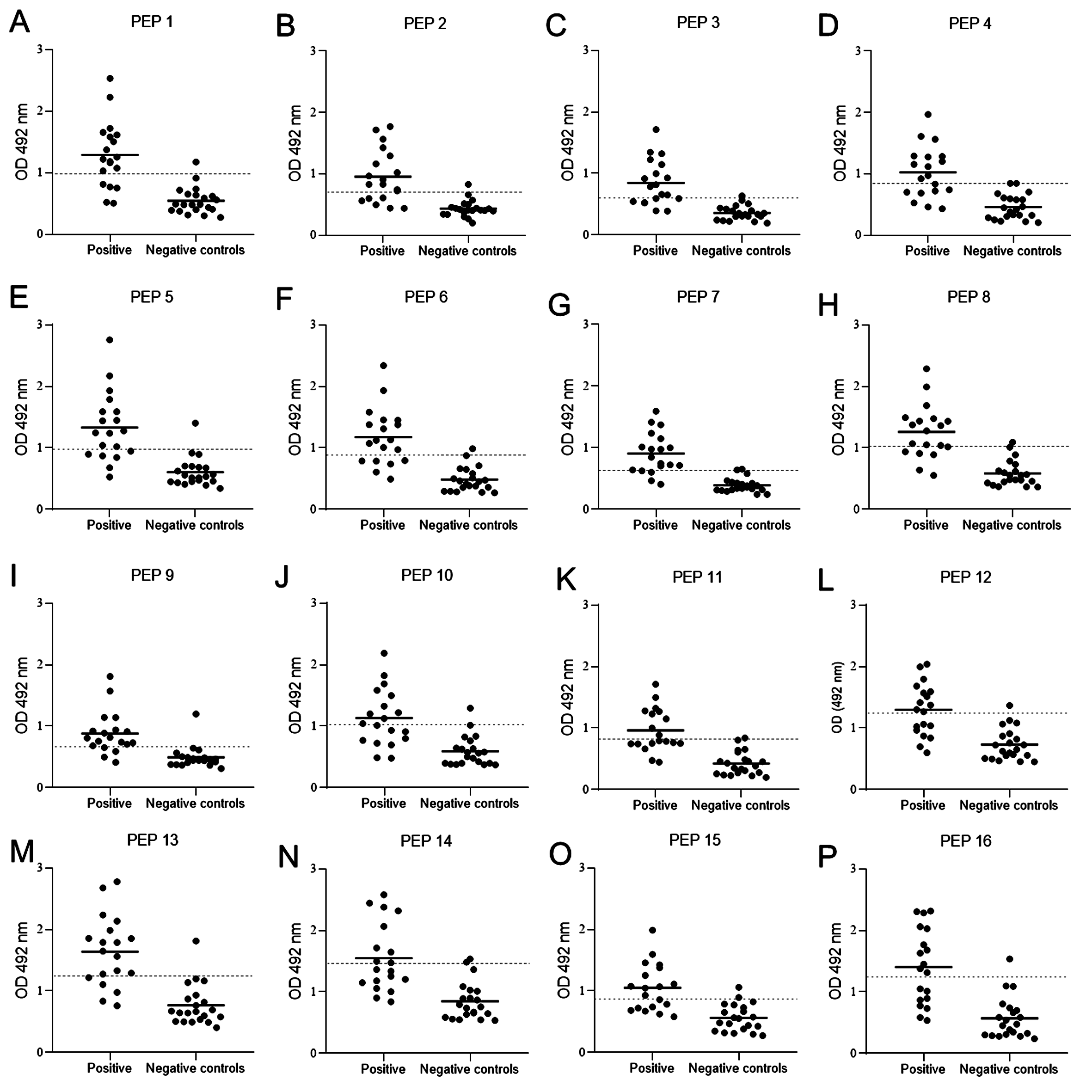

Supplement: S2 Fig — Data are presented as individual values for each positive control (n = 19) and negative control (n = 21) sample. The dashed line represents the cut-off point of each analysis, and the bar represents the mean OD of each group (Sensitivity, specificity, and OD ratio results are available in S2 Table). Peptides PEP1 (A), PEP2 (B), and PEP7 (G) were selected for pool based on the ROC curve results. All analyses were performed in duplicate, and the values represent the mean OD of each sample. (TIF) [file pone.0352788.s002.tif]

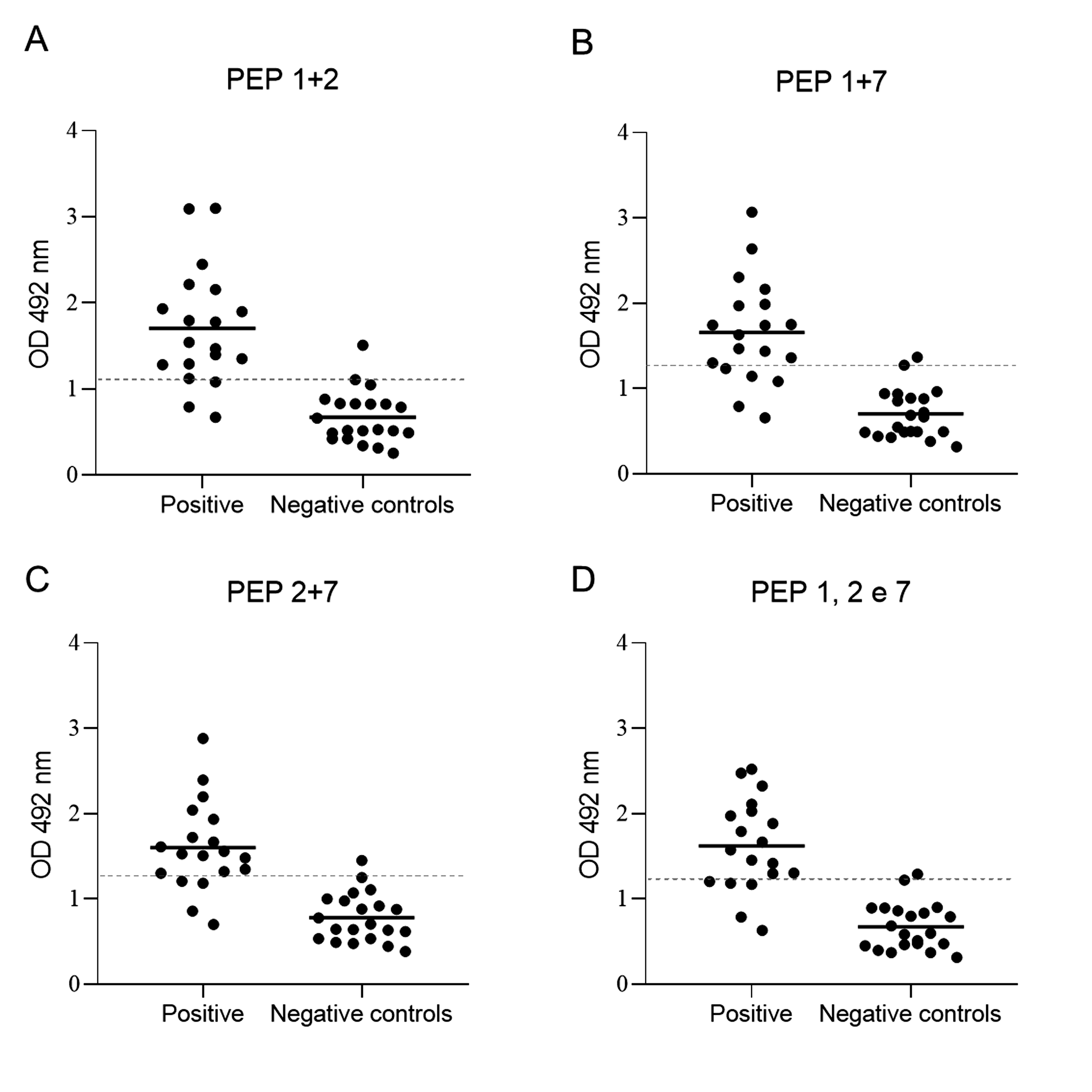

Supplement: S3 Fig — Data are presented as individual values for each positive (n = 19) and negative (n = 21) control sample. The dashed line represents the cutoff point for each analysis, and the bar represents the median OD of each group. A) PEP1 + PEP2 peptide pool showed superior results to the others based on ROC curve analysis, with sensitivity (84.21% − 95% CI = 62.43–94.48), specificity (95.24% − 95% CI = 77.33–99.76), and AUC (0.9373–95% CI = 0.8644–1.0) p < 0.0001. Sensitivity, specificity, and OD ratio results are available in S2 Table. All analyses were performed in duplicate, and the values represent the mean OD of each sample. (TIF) [file pone.0352788.s003.tif]
