# Supplementary material for: In silico-predicted B-cell epitopes for bovine brucellosis serodiagnosis: Preliminary analytical evaluation of synthetic peptide- and multi-epitope protein-based indirect ELISAs
Source: PLoS One. 2026 Jun 30;21(6):e0352788. doi: 10.1371/journal.pone.0352788 (PMC13318027; doi:10.1371/journal.pone.0352788)
Supplement: S1 Table — The columns represent the selected peptides P1-P16 and the densitometry values obtained in the sample pools of positive control, negative control, Leptospira spp., multiple infections, vaccinated with the RB51 vaccine at 45 days (45d) and 3 months post-vaccination, and vaccinated with S19 at three months (3m) and twelve months (12m) post-vaccination. Cut-off of 14640.81. (PDF) [file pone.0352788.s004.pdf]

S1 Table. **Densitometry values obtained for each selected peptide in the spot membrane immunoblotting technique.** The columns represent the selected peptides P1-P16 and the densitometry values obtained in the sample pools of positive control, negative control, *Leptospira* spp., multiple infections, vaccinated with the RB51 vaccine at 45 days (45d) and 3 months post-vaccination, and vaccinated with S19 at three months (3m) and twelve months (12m) post-vaccination. Cut-off of 14640.81.

Peptide	Positive control	Negative control	*Leptospira* spp.	Multiple infections	RB51 (45d)	RB51 (3m)	S19 (3m)	S19 (12m)
P 1	21628	2314	5335	7918	7324	12686	3955	5336
P 2	20011	3876	6794	9577	2377	11690	7676	3113
P 3	19386	10402	11526	6951	6490	12023	1524	2242
P 4	18134	7113	6352	2499	6184	6076	1360	1571
P 5	17148	2038	3817	9233	1803	14077	5277	1040
P 6	16649	8414	11997	5049	6122	8791	1596	2910
P 7	16083	9319	12128	10949	5103	13620	8978	5393
P 8	14736	3525	6412	7276	2425	14605	4592	1920
P 9	30223	9513	11313	9129	1739	18355	6235	2088
P 10	26373	4141	8101	10366	3643	18736	7834	882
P 11	24450	6031	5972	11385	2867	18540	5416	2294
P 12	23838	8030	13380	11612	7417	16308	10373	6188
P 13	23656	4847	8267	10542	3544	16666	6774	1374
P 14	23302	8392	9197	9017	4751	17792	4953	5964
P 15	21655	5523	13596	9798	9790	16554	3426	4807
P 16	21173	6182	8303	13588	4295	17758	8446	4036
