# Supplementary material for: In silico-predicted B-cell epitopes for bovine brucellosis serodiagnosis: Preliminary analytical evaluation of synthetic peptide- and multi-epitope protein-based indirect ELISAs
Source: PLoS One. 2026 Jun 30;21(6):e0352788. doi: 10.1371/journal.pone.0352788 (PMC13318027; doi:10.1371/journal.pone.0352788)
Supplement: S2 Table — (PDF) [file pone.0352788.s005.pdf]

S2 Table. Results of ROC curve analysis and OD ratio (positive control/negative control) of ELISAi of individual and pool peptides. p-value <0.0001 for all analyses.

**ELISA for individual peptides**					
Peptide	AUC	Sensitivity	Specificity	*Cut off*	OD Ratio
PEP 1	0,9323 (IC 95% = 0,8644- 1,0)	95,24% (IC 95% = 77,33 – 99,76)	73,68% (IC 95% = 51,21 -88,19)	0,9698	2,37
PEP 2	0,9261 (IC 95% = 0,8499 – 1,0)	95,24% (IC 95% = 77,33 – 99,76)	68,42% (IC 95% = 46,01 – 84,64)	0,6933	2,19
PEP 3	0,9411 (IC 95% = 0,8736 – 1,0)	95,24% (IC 95% = 77,33 – 99,76)	68,42% (IC 95% = 46,01 – 84,64)	0,5933	2,35
PEP 4	0,9023 (IC 95% = 0,8111 – 0,9934)	95,24% (IC 95% = 77,33 – 99,76)	57,89% (IC 95% = 36,28 – 76,86)	0,8465	2,28
PEP 5	0,9223 (IC 95% = 0,8397 – 1,0)	95,24% (IC 95% = 77,33 – 99,76)	68,42% (IC 95% = 46,01 – 84,64)	0,9842	2,19
PEP 6	0,9449 (IC 95% = 0,8807 – 1,0)	95,24% (IC 95% = 77,33 – 99,76)	68,42% (IC 95% = 46,01 – 84,64)	0,9244	2,41
PEP 7	0,9599 (IC 95% = 0,9082 – 1,0)	95,24% (IC 95% = 77,33 – 99,76)	78,95% (IC 95% = 56,67 – 91,49)	0,6435	2,30
PEP 8	0,9373 (IC 95% = 0,8654 – 1,0)	95,24% (IC 95% = 77,33 – 99,76)	68,42% (IC 95% = 46,01 – 84,64)	1,030	2,14
PEP 9	0,9073 (IC 95% = 0,8007 – 1,0)	95,24% (IC 95% = 77,33 – 99,76)	78,95% (IC 95% = 56,67 – 91,49)	0,6689	1,78
PEP 10	0,8697 (IC 95% = 0,7596 – 0,9797)	95,24% (IC 95% = 77,33 – 99,76)	52,63% (IC 95% = 31,71 – 72,67)	1,030	1,90
PEP 11	0,9223 (IC 95% = 0,8392 – 1,0)	95,24% (IC 95% = 77,33 – 99,76)	47,37% (IC 95% = 27,33 – 68,29)	0,8239	2,25
PEP 12	0,8722 (IC 95% = 0,7660 – 0,9784)	95,24% (IC 95% = 77,33 – 99,76)	57,89% (IC 95% = 36,28 – 76,86)	1,198	1,76
PEP 13	0,9248 (IC 95% = 0,8446 – 1,0)	95,24% (IC 95% = 77,33 – 99,76)	73,68% (IC 95% = 51,21 -88,19)	1,248	2,12
PEP 14	0,8847 (IC 95% = 0,7820 – 0,9874)	90, 48% (IC 95% = 71,09 – 98,31%)	47,37% (IC 95% = 27,33 – 68,29)	1,422	1,82
PEP 15	0,8822 (IC 95% = 0,7822 – 0,9822)	95,24% (IC 95% = 77,33 – 99,76)	57,89% (IC 95% = 36,28 – 76,86)	0,9136	1,86
PEP 16	0,8922 (IC 95% = 0,7957 – 0,9888)	95,24% (IC 95% = 77,33 – 99,76)	57,89% (IC 95% = 36,28 – 76,86)	1,208	2,44
**PEPTIDE POOL ELISAi**					
Peptide	AUC	Sensitivity	Specificity	*Cut off*	OD Ratio
PEP1+PEP2	0,9373 (IC 95% = 0,8644 – 1,0)	95,24% (IC 95% = 77,33 – 99,76)	84,21% (IC 95% = 62,43 – 94.48)	1,114	2,53
PEP1+PEP7	0,9323(IC 95% = 0,8561 – 1,0)	95,24% (IC 95% = 77,33 – 99,76)	73,68% (IC 95% = 51,21 -88,19)	1,289	2,35
PEP2+PEP7	0,9323 (IC 95% = 0,8549 – 1,0)	95,24% (IC 95% = 77,33 – 99,76)	78,95% (IC 95% = 56,67 – 91,49)	1,277	2,05
PEP1, PEP2 e PEP7	0,9373 (IC 95% = 0,8641 – 1,0)	95,24% (IC 95% = 77,33 – 99,76)	73,68% (IC 95% = 51,21 -88,19)	1,257	2,40
